# The characteristics of women not-attending screening and their barriers to attendance in Czechia

**DOI:** 10.1093/eurpub/ckac129.305

**Published:** 2022-10-25

**Authors:** A Altová, M Lustigová, I Kulhánová

**Affiliations:** Faculty of Science, Charles University, Praha, Czechia

## Abstract

**Background:**

Cervical screening is one of the most important tools to reduce the incidence and mortality of cervical cancer, but uptake is still insufficient in many European countries with implemented population screening programs. In this study, we analyze the sociodemographic characteristics of women who do not attend cervical screening and describe the barriers that these women may have to attend cervical screening using Czechia as an example.

**Methods:**

In the representative sample of 902 Czech women, we first employed binary logistic regression to identify groups of women that have higher chances of being non-attendees. Second, we described the reasons non-attendees declared as barriers to attendance. Third, we analyzed whether there were differences in women's characteristics according to the declared barriers.

**Results:**

In the study sample, 36.7% of the women were considered non-attendees. Women with lower education (primary compared to university education, OR = 2.2, 95%CI 1.2-3.9) single women (compared to married/partnered, OR = 3.6, 95%CI 2.0-5.1), or older women, had a higher chance of not attending the screening. The most frequently declared reasons for not attending were ‘not experiencing any symptoms’ (36.3%), ‘fear of cancer diagnosis’ (23.0%), and ‘fear of the examination procedure’ (20.2%). In most of these barriers, women declaring these reasons did not differ from the other non-attendees.

**Conclusions:**

Identifying sociodemographic determinants of cervical screening non-attendance and the barriers women have to attend are crucial for improving cervical cancer prevention. Based on this knowledge, public health policies should minimize screening hesitancy by targeting psychological factors and improving screening literacy among women. Although this research is a case study for Czechia, we believe that the results may be applicable in other countries.

**Key messages:**

• Cervical screening promotion should be targeted on women at higher risk of non-attendance.Those are women with lover education, single women or older women.

• Understanding the specific barriers to cervical screenin attendance could help develop strategies to improve the communication of prevention.

